# The Use of Extracorporeal Membrane Oxygenation in Traumatic Brain Injury and Neurosurgical Patients: A Single-Center Analysis and Systematic Review

**DOI:** 10.1007/s12028-026-02449-8

**Published:** 2026-02-26

**Authors:** Raahim Bashir, Hardik Bhaskar, Justin Oh, Christopher Tanski, Timothy Beutler

**Affiliations:** 1 Department of Neurosurgery, University of Missouri–Columbia, Columbia, MO USA; 2 Norton College of Medicine, SUNY Upstate Medical University, Syracuse, NY USA; 3 Department of Neurosurgery, SUNY Upstate Medical University, Syracuse, NY USA; 4 Department of Emergency Medicine, SUNY Upstate Medical University, Syracuse, NY USA

**Keywords:** Traumatic brain injury, Extracorporeal membrane oxygenation, Intracranial hemorrhage, Neurosurgical intervention

## Abstract

**Background:**

This study evaluates the outcomes and management considerations associated with using extracorporeal membrane oxygenation (ECMO) in patients with traumatic brain injury (TBI), including those requiring neurosurgical intervention. It examines the feasibility of managing patients with TBI and neurosurgical patients on ECMO by synthesizing institutional data with the existing literature, emphasizing observed clinical contexts, anticoagulation strategies, and peri-procedural factors relevant to safe ECMO use. Our aim is to elucidate the potential for ECMO to be safely applied in patients with TBI needing intensive cardiopulmonary support, and for neurosurgical intervention to remain an option when indicated.

**Methods:**

We retrospectively reviewed the medical records of seven patients with TBI who received ECMO therapy at our institution. The peri-ECMO period was defined as 7 days before ECMO cannulation to 7 days after decannulation. A systematic review of the literature was conducted to compare outcomes, complications, and management strategies.

**Results:**

Most patients in our cohort were managed without systemic anticoagulation during ECMO treatment. Five patients underwent neurosurgical procedures in the peri-ECMO period with overall positive outcomes. In the systematic review, outcomes for patients with TBI receiving ECMO varied; while some demonstrated neurological improvement, others succumbed to complications such as septic shock and multiorgan failure. A subset of patients with TBI developed intracranial hemorrhage (ICH) while on ECMO, a concern that often discourages its use. They, along with other non-TBI patients who developed ICH while on ECMO, were subsequently treated with neurosurgical interventions with the majority showing functional improvement. Key factors affecting prognosis included ICH size, timing of neurosurgical intervention, and careful adjustment of anticoagulation therapy.

**Conclusions:**

This study highlights that ECMO can be safely utilized in patients with TBI requiring intensive cardiopulmonary support, including those undergoing neurosurgical procedures for ECMO-related complications. In this high-risk population, maintaining ECMO circuits without systemic anticoagulation or with conservative anticoagulation should be considered. Our findings suggest that TBI should not be an absolute contraindication for ECMO therapy, and neurosurgical interventions can be safely performed in these patients, especially when ICH occurs.

## Introduction

Traumatic brain injury (TBI) is a leading cause of mortality and long-term disability worldwide, with more than 235,000 annual TBI-related hospitalizations in the USA alone [1]. Patients with severe TBI often have prolonged intensive care unit (ICU) admissions and are complex to manage due to accompanying polytrauma and multiple organ dysfunction. These patients frequently need prolonged mechanical ventilation and are at elevated risk for acute respiratory distress syndrome (ARDS) [2]. The prevalence of ARDS among patients with TBI is approximately 19%, arising secondary to factors such as noncardiogenic pulmonary edema, ventilatory lung injury, or coexisting thoracic trauma [3]. Standard ARDS management with lung-protective ventilation (LPV) with low tidal volume and high positive end-expiratory pressure (PEEP) ventilation strategies permit hypercapnia to avoid ventilator-associated lung injury [2]. In TBI, however, permissive hypercapnia and increased intrathoracic pressure can exacerbate intracranial pressures (ICP) [4]. This creates a challenging dilemma: LPV strategies beneficial to ARDS may worsen ICP control. This is where traditional goals of managing posttraumatic respiratory failure, such as preventing barotrauma and ventilator-associated lung injury, may be paradoxically detrimental for the patient wtih TBI [5]. While TBI does not preclude LPV, there is no uniform consensus on optimal respiratory management of patients with severe TBI with concurrent respiratory failure [6].

Extracorporeal membrane oxygenation (ECMO) is a rescue strategy for refractory hypoxemia, respiratory failure, or cardiogenic shock. ECMO works by diverting blood through an external circuit for gas exchange, thereby supporting oxygenation and CO_2_ removal independent from lung function [7]. Although a lifesaving intervention, ECMO requires systemic anticoagulation. Anticoagulation is commonly achieved with heparin, which is administered to prevent clotting of the ECMO circuit, which can lead to pulmonary emboli (PE), myocardial infarction (MI), and ischemic stroke. Heparinization is associated with serious complications including thrombosis, hemolysis, bleeding, and neurological injury (especially intracerebral hemorrhage) [8]. In fact, ICH has been reported to occur in approximately 10–15% of patients with ARDS on ECMO, accounting for a significant portion of ECMO-related mortality [7]. Consequently, severe TBI is often considered a relative contradiction to ECMO due to the risk of precipitating or worsening ICH. Further, the presence of ARDS in patients with TBI is known to decrease survival and positive neurological outcomes and warrants further investigation into the use of ECMO in this population [3].

Therefore, we present a single-center retrospective case series of patients with severe TBI who underwent ECMO therapy during their hospitalization, focusing on clinical decision-making, anticoagulation management, functional outcomes, and complications. In addition, we performed a comprehensive systematic review to contextualize our findings. We examined reported cases of ECMO use in TBI and cases where neurosurgical procedures were performed for ECMO-related intracranial complication. Through this study, we aim to provide updated insights into the feasibility, safety, and outcomes with using ECMO in patients with severe TBI.

## Methods

### Institutional Cohort

A single-center retrospective case series evaluating seven patients with severe TBI who received ECMO therapy at our institution was performed using electronic medical records (EMR). The peri-ECMO period was defined as 7 days before ECMO cannulation to 7 days after decannulation. Data collected included patient demographics, ECMO parameters, anticoagulation strategies, neurosurgical interventions, and outcomes (Table 1). We specifically noted any intracranial complications occurring during ECMO support. All neuroimaging findings (e.g., cerebral contusions, hematomas, diffuse axonal injury) were identified on initial head computed tomography (CT) scans, with magnetic resonance imaging (MRI) being used in select cases as clinically indicated. The study was approved by the Institutional Review Board as an exempt study.

**Table 1:** Patients with TBI undergoing ECMO (institutional case series)

Age (years)	Sex	Initial GCS	Type of TBI	Indication for ECMO	Number of days from TBI to ECMO	GCS at ECMO cannulation	ECMO type	Anticoagulation	Anticoagulation dose and profile	Neurosurgical intervention	Total duration on ECMO (Days)	Outcome
25	M	11	EDH; SAH; contusion	ARDS	22	6	VV	Heparin	5000 U, aPTT of 40–60 s	DHC at 9 days prior ECMO	21	GCS 15, neuro intact
25	M	3	SAH; contusion	ARDS	3	6	VV	None	N/A	DHC while on ECMO Day 0	7	GCS 15, neuro intact
16	M	3	SDH; SAH; contusion; DAI	ARDS, refractory hypoxemia/hypercarbia, cardiac arrest	0	3	VV	None	N/A	Decompressive craniectomy and SDH evacuation on ECMO day 3	4	Died
32	M	3	DAI	Acute hypoxemic respiratory failure, cardiac arrest	2	3	VA	Heparin	10,000 U bolus, 1000 U/h infusion, aPTT of 60–80 s	N/A	2	At discharge: GCS 15, neuro intact
16	M	14	EDH; contusion; SDH; DAI	ARDS, refractory hypoxemia/hypercarbia	3	6	VV	None	N/A	Craniotomy and EDH evacuation 2 days prior to ECMO	3	GCS 15, MRS = 1–2, residual weakness
13	M	5	SDH	ARDS, refractory hypoxemia/hypercarbia, cardiac arrest	0	5	VV	None	N/A	N/A	3	GCS 15, neuro intact
36	M	13	SDH; SAH; contusion	ARDS, acute hypoxemic respiratory failure	10	10	VV	Heparin	5000 U bolus, 2700 U/h infusion, aPTT of 100–150 s	Developed IPH 5 days after ECMO decannulation secondary to therapeutic anticoagulation for PE; DHC with IPH evacuation	6	GCS 14

*aPTT* activated partial thromboplastin time, *ARDS* acute respiratory distress syndrome, *DAI* diffuse axonal injury, *DHC* decompressive hemicraniectomy, *ECMO* extracorporeal membrane oxygenation, *EDH* epidural hematoma, *GCS* Glasgow coma scale, *IPH* intraparenchymal hematoma, *M* male, *MRS* modified Rankin scale, *PE* pulmonary embolism, *neuro* neurologically, *N*/*A* not applicable, *SAH* subarachnoid hemorrhage, *SDH* subdural hematoma, *TBI* traumatic brain injury, *U* units, *VV* veno-venous, *VA* veno-arterial

### Systematic Review

**Fig. 1:**
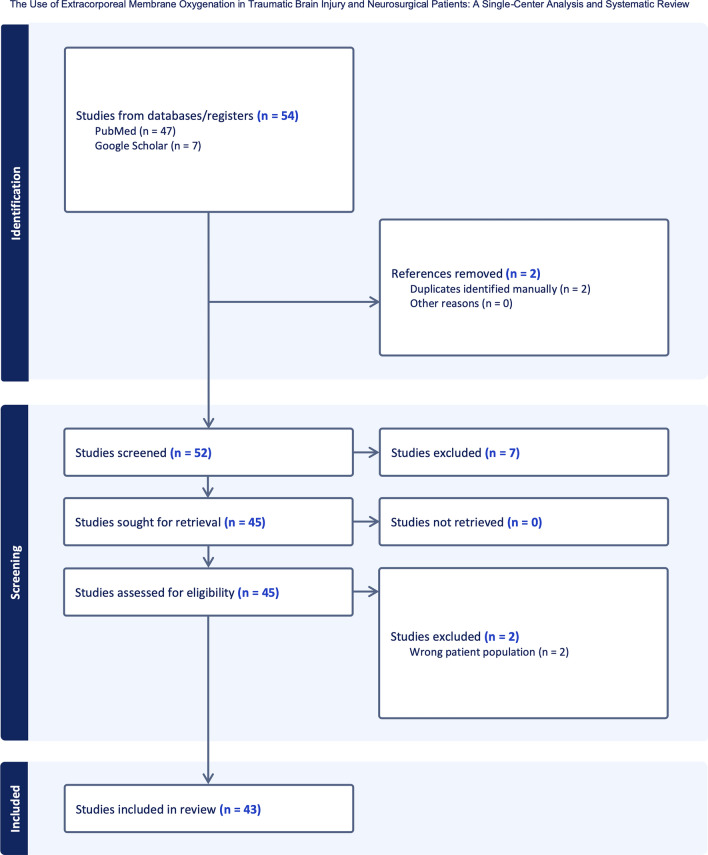
PRISMA flow diagram illustrating the study selection process. A total of 54 records were identified through database searching (PubMed: *n* = 47; Google Scholar: *n* = 7). After removing 2 duplicates, 52 studies were screened; 7 were excluded on the basis of title and abstract, and 2 additional studies were excluded after full-text review due to incorrect patient population. Ultimately, 43 studies were included in the final review

A comprehensive PROSPERO registered (CRD42024610714) systematic review was conducted according to Preferred Reporting Items for Systematic Reviews and Meta-Analyses (PRISMA) guidelines. A systematic search of PubMed and Google Scholar without language or date restrictions was designed to comprehensively identify studies relevant to the use of ECMO in patients with TBI and those undergoing neurosurgical interventions. The search was conducted using two sets of terms. The first set combined terms for ECMO and neurosurgical procedures using Boolean operators, specifically using the terms: [“extracorporeal membrane oxygenation” OR “ECMO”] AND [“craniectomy,” OR “craniotomy,” OR “decompressive craniectomy,” OR “decompressive hemicraniectomy”]. The second set targeted ECMO and TBI using Boolean operators, specifically using the terms: [“extracorporeal membrane oxygenation” OR “ECMO”] AND [“traumatic brain injury” OR “TBI”]. To enhance the search’s comprehensiveness, references from identified studies were screened manually for additional relevant publications. This strategy ensured the inclusion of both indexed and non-indexed studies pertinent to the topic. Two independent reviewers screened titles, abstracts, and full-text articles against predefined eligibility criteria. Disagreements were resolved by consensus or a third reviewer. Studies were included if they: (1) reported on ECMO use in patients with TBI (Tables 2 and 4) or (2) described patients undergoing neurosurgical interventions to treat an intracranial complication suffered while on ECMO (Table 3). Exclusion criteria included studies on ECMO patients that did not specifically mention TBI (Tables 2 and 4), studies in which patients received ECMO only after a neurosurgical procedure for nontraumatic indications was performed [(e.g., ECMO initiated for neurogenic pulmonary edema after cerebral aneurysm clipping) (Table 3)], and nonhuman studies. Additionally, studies in which patients underwent only bedside procedures, such as placement of an ICP monitor or an external ventricular drain (EVD), were excluded, as our focus was on more invasive operative interventions. Studies in which patients received any other means of extracorporeal life support other than ECMO, such as interventional lung assist, cardiopulmonary bypass, extracorporeal lung assist, and left ventricular assist devices, were also excluded. A totalfo 54 papers were initially screened, of which 43 papers were included for analysis (Fig. 1); these were then extrapolated into three different datasets that included patients with TBI undergoing ECMO on the basis of case reports/series (Table 2), case reports/series on patients receiving neurosurgical intervention for an ECMO-related complication (Table 3), and patients with TBI undergoing ECMO on the basis of cohort and review studies (Table 4).

**Table 2:** Patients with TBI undergoing ECMO (case reports and series)

Study	Age (years)	Sex	Initial GCS	ISS	Type of TBI	Indication for ECMO	Number of days from TBI to ECMO	GCS at ECMO	ECMO type	Anticoagulation used	Anticoagulation dose and coagulation profile target on ECMO	Intracranial complication of ECMO	If intracranial complication encountered, was ECMO discontinued?	If intracranial complication encountered, was anticoagulation discontinued?	Neurosurgical intervention	Total duration on ECMO (Days)	Outcome
Frickey et al. 2008 [42]	41	M	8	N/A	EDH; Contu-sion; ICH	Cardiac arrest	5	N/A	VA	Heparin	Not specified	None	N/A	N/A	None	7	Hospital day 48: normal neuro exam; 1-year follow-up: MRS 0–1
Leloup et al. 2011 [43]	27	M	N/A	N/A	ICH	ARDS	N/A	N/A	VV	Not specified	Not specified	None	N/A	N/A	None	7	At discharge: normal neuro status
Stoll et al. 2014 [32]	18	M	N/A	51	ICH	Refractory hypoxemia/hypercarbia	1	N/A	VV	Heparin initiated after 72 h of initial ECMO with no anticoagulation	Low dose of 200 IU	None	N/A	N/A	None	~ 10 days	Good recovery
Friese-necker et al. 2005 [30]	34	M	11	N/A	Contu-sion	ARDS; refractory hypoxemia/hypercarbia	2	8	VV	Heparin	Dose not specified; ACT of 150 s	R ICH on ECMO day 2	No	No	Burr hole drainage	17	At discharge: GCS 8 T; 6-month follow-up: GCS 11
Anton-Marton et al. 2018 [5]	8	F	12	N/A	Conc-ussion	ARDS; refractory hypoxemia/hypercarbia	5	N/A	VV	Heparin	maximum drip rate of 20 U/kg/h; ACT of 160–170 s and aPTT of 70–80 s	R IPH, IVH, 12 mm midline shift, sulcal and cisternal effacement, on ECMO day 7	Yes	Discontinued and reversed with protamine	Decompressive craniectomy with hematoma evacuation	7	At 6-month follow-up: GCS 15 and MRS 0–1
Bindal et al. 2022 [11]	18	F	~ 3–8	N/A	SAH	Cardiac arrest	Same day	~ 3–8	VA	Heparin	5000 U	R IPH on ECMO day 1	No	Discontinued and reversed with protamine	DHC with partial hematoma evacuation, extension of the DHC and further evacuation the next day	2 (decann-ulated after second DHC)	At discharge (hospital day 19): GCS 15; 3-month follow-up: MRS 0
Zhou et al. 2015 [44]	31	M	N/A	N/A	SAH; contu-sion	Refractory hypoxemia/hypercarbia	N/A	N/A	VA	Heparin	1 mg/kg; initial ACT of 300 s, maintained at 140–180 s	None	N/A	N/A	None	0.38	Patient lived normal life; neuro intact
Messing et al. 2014 [45]	21	M	N/A	38	SDH; SAH; DAI	ARDS; refractory hypoxemia/hypercarbia	3	N/A	VV	Heparin	Dose not specified; ACT of 160–180 s	None	N/A	N/A	None	20	At unspecified follow-up: MRS 0, GCS 15
Yen et al. 2008 [46]	21	M	12	N/A	SDH; EDH	V. tach; refractory hypoxemia/hypercarbia	~ 2 (ECMO initiated on second postop day)	3–8	VA	None	N/A	None	N/A	N/A	Decompressive craniotomy and hematoma evacuation 2 days before ECMO	2	At discharge: full neuro recovery
Trivedi et al. 2021 [47]	24	M	N/A	N/A	ICH	ARDS	9	N/A	VV	None	N/A	None	N/A	N/A	None	17	Hospital survival
Robba et al. 2017 [27]	58	M	6	N/A	SDH; contu-sion	Refractoryhypoxemia/hypercarbia	5	N/A	VV	Heparin	5000 U	None	N/A	N/A	None	15	At discharge: GCS 15
Robba et al. 2017 [27]	54	M	4	N/A	SDH;SAH; contu-sion	Hypoxemic respiratory failure	10	N/A	VV	Heparin	500 U	None	N/A	N/A	None	4	At discharge: deceased
Muelle-nbach et al. 2012 [47]	53	M	4	59	SAH; contu-sion	ARDS; refractory hypoxemia/hypercarbia	~ 1	N/A	VV	None for first 5 days, low-dose heparin on ECMO day 5	Dose not specified: aPTT of 40–50 s	None	N/A	N/A	None	8	FIM: 68/126 points after rehab
Muelle-nbach et al., 2012 [47]	16	M	3	66	SDH	ARDS; refractory hypoxemia/hypercarbia	~ 0.5	N/A	VV	None for first day, low-dose heparin on ECMO day 2	Dose not specified: aPTT of 40–50 s	None	N/A	N/A	None	~ 3	Resumed school 6 months post-trauma
Muelle-nbach et al. 2012 [47]	28	M	6	66	Contu-sion	ARDS; refractory hypoxemia/hypercarbia	In ER on admit	N/A	VV	None	N/A	None	N/A	N/A	None	~ 3	FIM: 61/126 points after rehab
Biscotti et al. 2015 [48]	18	M	14	27	EDH; SAH; contu-sion	ARDS; refractory hypoxemia/hypercarbia	4	N/A	VV	Heparin	Low-dose anticoagulation protocol; aPTT of 40–60 s	None	N/A	N/A	None	13	Complete neuro recovery
Biscotti et al. 2015 [48]	20	M	3	33	SDH;SAH; contu-sion	ARDS	4	N/A	VV	Heparin	Low-dose anticoagulation protocol; aPTT of 40–60 s	None	N/A	N/A	None	6	Complete neuro recovery
Reynoldset al. 1999 [49]	16	M	6 T	N/A	Contu-sion	Refractory hypoxemia/hypercarbia	Same day	N/A	VV	None	N/A	None	N/A	N/A	None	6.67	At discharge: neuro intact, with only moderate cognitive deficits
Firsten-berg et al. 2012 [31]	27	M	N/A	N/A	SDH; ICH; IVH	Refractory hypoxemia/hypercarbia	On admit	N/A	VV	Heparin bolus at cannulation, followed by heparin infusion 48 h later	10,000 units bolus, 4.0 units/kg/h infusion	None	N/A	N/A	None	4	At discharge (post-trauma day 23): neuro intact
Bruzek et al. 2014 [50]	25	M	7 T	N/A	EDH	ARDS	10 (ECMO initiated on tenth postop day)	N/A	VV	Heparin	Not specified	None	N/A	N/A	Decompressive craniectomy and EDH evacuation 10 days before ECMO	7	6-month follow-up: GOS 4

*aPTT* activated partial thromboplastin time, *ACT* activated clotting time, *ARDS* acute respiratory distress syndrome, *DAI* diffuse axonal injury, *DHC* decompressive hemicraniectomy, *ECMO* extracorporeal membrane oxygenation, *EDH* epidural hematoma, *F* female, *FIM* functional independence measure, *GCS* Glasgow coma scale, *GOS* Glasgow outcome scale, *ICH* intracranial hemorrhage, *IPH* intraparenchymal hematoma, *IVH* intraventricular hemorrhage, *M* male, *MRS* modified Rankin scale, *neuro* neurologically, *N*/*A* not applicable*SAH* subarachnoid hemorrhage, *SDH* subdural hematoma, *TBI* traumatic brain injury, *U* units, *VV* veno-venous, *VA* veno-arterial,

**Table 3:** Patients receiving neurosurgical intervention for an ECMO-related complication

Study	Age	Sex	Initial GCS	Indication for ECMO	GCS at ECMO	ECMO type	Anticoagulation used	Anticoagulation dose and coagulation profile target on ECMO	Intracranial complication of ECMO	If intracranial complication encountered, was ECMO discontinued?	If intracranial complication encountered, was anticoagulation discontinued?	Neurosurgical intervention	Total Duration on ECMO (Days)	Outcome
Friesenecker et al. 2005 [30]	34 years	M	11	ARDS; refractory hypoxemia/hypercarbia	8	VV	Heparin	Dose not specified; ACT of 150 s	R ICH on ECMO day 2	No	No	Burr hole drainage	17	At discharge: GCS 8 T; 6-month follow-up: GCS 11
Anton-Marton et al. 2018 [5]	8 years	F	12	ARDS; refractory hypoxemia/hypercarbia	N/A	VV	Heparin	Maximum drip rate of 20 U/kg/h; ACT of 160–170 s and aPTT of 70–80 s	R IPH, IVH, 12 mm midline shift, sulcal and cisternal effacement, on ECMO day 7	Yes	Discontinued and reversed with protamine	Decompressive craniectomy with hematoma evacuation	7	At 6-month follow-up: GCS 15 and MRS 0–1
Bindal et al. 2022 [11]	18 years	F	~ 3–8	Cardiac arrest	~ 3–8	VA	Heparin	5000 U	R IPH on ECMO day 1	No	Discontinued and reversed with protamine	DHC with partial hematoma evacuation, extension of the DHC and further evacuation the next day	2 (decannulated after 2nd DHC)	At discharge (hospital day 19): GCS 15; 3-month follow-up: MRS 0
Factora et al. 2011 [51]	43 years	F	N/A	Cardiac arrest and myocardial infarction during heart surgery	N/A	VA	None	N/A	R hemorrhagic infarct on ECMO day 4	No	Not specified	Decompressive hemicraniotomy	~ 4 (decannulated same day as neurosurgical intervention)	Patient expired ~ 3 days after decannulation (MRS 6)
Kraus et al. 2015 [52]	38 years	M	N/A	ARDS	N/A	VV	Heparin	5000 U bolus followed by 1000 U/h infusion; initial ACT of 160 – 220 s, protamine 20 mg administered intraoperatively to maintain an ACT of 141 s	R IPH with midline shift and downward uncal herniation on ECMO day 5	No	Yes	R craniotomy and clot evacuation, second craniotomy 1 day after first	9.875	Care withdrawn, patient not expected to survive
Jolly et al. 2023 [53]	39 years	F	N/A	ARDS	N/A	VV	Yes (type not specified	Not specified	Anticoagulation withdrawn due to GI bleed, subsequently patient developed cerebral venous sinus thrombosis for which heparin infusion started, subsequently patient developed SAH, IVH, and posterior fossa SDH	No	Not specified	Suboccipital craniotomy with posterior fossa hematoma evacuation	Not specified	Initial postoperative neuro improvements followed by unsatisfactory recovery necessitating comfort care
Horio et al. 2023 [32]	54 years	M	N/A	Cardiac arrest; cardiogenic shock	N/A	VA	Heparin	12,000 U total	R ICH 19 days after ECMO decannulation	Decannulated 19 days prior to intracranial complication	Already discontinued with ECMO decannulation	Surgical hematoma evacuation	7	4 months after discharge: MRS 4–5
Horio et al. 2023 [33]	52 years	M	N/A	Metabolic acidosis; multiple organ failure; hemodynamic instability	N/A	VA	Heparin	12,000 U total	L ICH after 3 days on ECMO	No	Discontinued and reversed with protamine	Surgical hematoma evacuation	14	Hospital day 14: MRS 6 (death)
Lilly et al. 2024 [28]	54 years	M	N/A	Acute hypoxic respiratory failure; sepsis; cardiac arrest; cardiogenic shock	N/A	VV	None	N/A	R MCA stroke with hemorrhagic transformation 12 h after ECMO decannulation on hospital day 20	Decannulated 12 h prior to intracranial complication	N/A	R DHC with R anterior temporal lobectomy and ICH evacuation	17	At 1 year: MRS 3
Lilly et al. 2024 [28]	39 years	F	N/A	Acute hypoxic respiratory failure	N/A	VV	Heparin	Not specified	Posterior fossa SDH after 42 days on ECMO	No (Decannulated on postoperative day 2 after neurosurgical intervention)	Discontinued and reversed with protamine	Decompressive suboccipital craniectomy with SDH evacuation	44	At 1 year: MRS 6 (expired on hospital day 64)
Lilly et al. 2024 [28]	37 years	F	N/A	Acute hypoxic respiratory failure; cardiogenic shock	N/A	VA	Heparin	Not specified	R cerebellar ICH, bilateral occipital ICH after 5 days on ECMO	No (decannulated right after neurosurgical intervention)	Already discontinued after ECMO cannulation due to concern for ischemic stroke	Decompressive suboccipital craniectomy with cerebellar ICH evacuation	6	At 1 year: MRS 6 (expired on hospital day 4)
Lilly et al. 2024 [28]	19 years	F	N/A	Cardiogenic shock	N/A	VA	Heparin, Eliquis	Not specified	L ICH with uncal herniation on hospital day 2 in immediate postop period after mitral valve revision (while on LVAD)	Decannulated on hospital day 2 and switched to LVAD prior to intracranial complication	Not specified	L craniotomy with ICH evacuation	1 (switched to LVAD)	At 1 year: MRS 6 (expired on hospital day 9)
Papin et al. 2019 [54]	38 years	M	N/A	Cardiogenic shock	< 8	VA	Heparin	Not specified	Cerebellar ICH on ECMO day 9	No	Yes	Craniotomy with hematoma evacuation	~ 14	90 days post-discharge: MRS 1
Sathyavadhi et al. 2021 [54]	41 years	M	N/A	ARDS; refractory hypoxemia/hypercarbia	N/A	VV	Heparin	Dose not specified; initial ACT of 180–220 s and aPTT of 60–80 s, pre-surgery ACT of 150 s and, aPTT of 30 s	R IPH and IVH, 17 mm midline shift, and effacement of suprasellar cisterns on ECMO day 3	No	Yes	R decompressive craniectomy	12	Expired on day 12 of ECMO due to septic shock and MODS
Fletcher-Sandersjöö et al. 2017 [12]	20 years	F	N/A	Cardiac arrest	N/A	VA	Heparin	Dose not specified; ACT of 204 s and aPTT of 55 s	IPH and SDH on ECMO day 6	No	Yes	SDH evacuation	11	At 6-month follow-up: GOS 5; full recovery within 1 year
Fletcher-Sandersjöö et al. 2017 [12]	35 years	F	N/A	Respiratory failure refractory to mechanical ventilation	N/A	VA	Heparin	Dose not specified; ACT of 200 s and aPTT of 50 s	Cerebellar IPH, SAH, IVH, and absent basal cisterns on ECMO day 32	No	Discontinued and reversed with protamine	Cerebellar hematoma evacuation	~ 32	Treatment withdrawn on day 33 and patient expired
Fletcher-Sandersjöö et al. 2017 [12]	67 years	M	N/A	Septic shock; respiratory failure; cardiac arrests	N/A	VA	Not specified	Dose not specified; aPTT of 30 s	Cerebellar IPH, parietal IPH, imminent cerebral herniation on ECMO day 6	Decannulated prior to intracranial complication	N/A	Cerebellar hematoma evacuation	6	30-day survival but suffered massive PE and expired few weeks later
Hervey-Jumper et al. 2011 [13]	3 months	M	N/A	ARDS	N/A	VA	Heparin	2 U/kg/h; ACT of 160–200 s	L SDH on ECMO day 8	No	No	Craniotomy with hematoma evacuation	~ 12.67	At 16-year follow-up: hemiparetic, cerebral palsy and complex partial seizure disorder
Hervey-Jumper et al. 2011 [13]	10 days	F	N/A	Acute respiratory failure; refractory hypoxemia/hypercarbia	N/A	VV	Heparin	2 U/kg/h; ACT of 160–180 s	L frontal IPH on ECMO day 7	No	Yes	DHC	~ 8	Treatment withdrawn on day 8 and patient expired
Hervey-Jumper et al. 2011 [13]	13 months	M	N/A	ARDS; refractory hypoxemia/hypercarbia	N/A	VA	Heparin	2 U/kg/h; ACT of 160–200 s	L cerebellar IPH on ECMO day 5	Yes	Discontinued and reversed with protamine and FFP	Suboccipital craniotomy with hematoma evacuation	5	At 10-year follow up: neuro intact
Young et al. 2024 [40]	3.5 years	F	N/A	Cardiac arrest	N/A	Not specified	Bivalirudin	Not specified	Ischemic cerebral edema (potentially exacerbated by ECMO)	Yes	Yes	DHC	~ 3	At 5-month follow up: mildly Hemiparetic, speaking single words

*aPTT* activated partial thromboplastin time, *ACT* activated clotting time, *ARDS* acute respiratory distress syndrome, *DAI* diffuse axonal injury, *DHC* decompressive hemicraniectomy, *ECMO* extracorporeal membrane oxygenation, *F* female, *GCS* Glasgow coma scale, *GI* gastrointestinal, *GOS* Glasgow outcome scale, *ICH* intracranial hemorrhage, *IPH* intraparenchymal hematoma, *IVH* intraventricular hemorrhage, *LVAD* left ventricular assist device, *MRS* modified Rankin scale, *TBI* traumatic brain injury, *M* male, *MCA* middle cerebral artery, *N*/*A* not applicable, *neuro* neurologically, *PE* pulmonary embolism, *SAH* subarachnoid hemorrhage, *SDH* subdural hematoma, *U* units, *VV* veno-venous, *VA* veno-arterial

**Table 4:** Patients with TBI undergoing ECMO (cohort studies and reviews). These data are representative of 882 patients across 17 studies, with an approximate average mortality of 30.2% across all studies

Study	Total patients with TBI and ECMO patients	Age range	Male	Female	VV ECMO type	VA ECMO type	Pre-ECMO assessment	Estimated average ISS	Initial GCS	ECMO indication	ECMO days	Neurosurgical intervention	Outcome (mortality)	Anticoagulation
Ahmad et al. 2016 [17]	11 (24% of ECMO Patients)	Median 31 (IQR 23.5–43.5)	72%	28%	85%	15%	Median ISS 33 (IQR 22–41)	31.50	NA	ARDS	Median 9.25 IQR 5–15	6 neurosurgical interventions	81.2% for TBI and ECMO	Yes, 27.3% heparinization
Al-Thani et al. 2022 [20]	15 (68.2% of ECMO Patients)	29.6 ± 13.8	86.40%	13.60%	95.50%	4.50%	Head AIS 3.9 ± 0.9	NA	9.5 (3–15)	ARDS due to trauma	Median 9.5 (1–29)	2 craniectomies	20% for TBI and ECMO	Yes, unspecified
Biderman et al. 2013 [18]	3 (60% of ECMO Population	Average 52.4	60%	40%	VV present (unspecified)	VA present (unspecified)	Average ISS 50.3	50.30	5 to 9	ARDS	7.6 days	NA	0% mortality for TBI and ECMO	No, heparin-free
Bosarge et al. 2016 [23]	2 (13.3% of ECMO Population)	36 (IQR 25–47)	100%	0%	VV present (unspecified)	VA present (unspecified)	Median ISS 26 (IQR 17–34)	25.50	NA	ARDS	8.1 days ± 5.2 days	NA	0% mortality for TBI and ECMO	Yes, low-dose heparin
Chen et al. 2016 [10]	4 (57.1% of ECMO Population	NA	86%	14%	100%	0%	Average ISS 41	0.41	NA	Acute lung injury	4 to 17 days	NA	25% mortality for TBI & ECMO	No, heparin-free
Guttman et al., 2020 [24]	82 (30.5% of ECMO Population)	Average 34.4	85.90%	14.10%	NA	NA	Average ISS 30.6	30.60	95% < 6	46% acute respiratory distress	NA	NA	32% ECMO mortality	NA
Hatfield et al. 2022 [15]	118 (100% Of ECMO Patients)	30 (22–46)	78%	22%	VV present (unspecified)	VA Present (Unspecified)	Average ISS 29	29.00	NA	ARDS	NA	NA	33.9% mortality	NA
Henry et al. 2021 [56]	48 (49.5% of ECMO Patients)	35 (22–51)	81%	19%	100%	0%	Median ISS 27 (IQR 17–34)	25.50	Median 14 (IQR 3–15)	ARDS	NA	NA	23% ECMO mortality	53% anticoagulation
Kruit et al. 2019 [19]	19 (37% of ECMO Population)	33 (IQR 24–45)	81%	19%	VV present (unspecified)	VA present (unspecified)	Median ISS of 35	35.00	NA	Respiratory contusion, ARDS	2 Days	NA	16% mortality for TBI and ECMO	63% heparinization
Mader et al. 2023 [21]	134 (100% of ECMO Population)	Average 49.8	80%	20%	VV present (unspecified)	VA present (unspecified)	Average ISS 35.9	35.90	53% < 8	ARDS	NA	59 (44%) neurosurgical interventions	38% mortality	NA
Munoz-Bendix et al. 2015 [2]	1 ECMO case (10% of TBI Cohort)	Average 53	0%	100%	100%	0%	NA	NA	NA	ARDS, heart failure	6 Days	NA	0% mortality for TBI and ECMO	NA
Parker et al. 2020 [22]	13 (100% of ECMO Population)	28 (IQR 25–37)	85%	15%	100%	0%	Median ISS 48 (IQR 33.5–66)	49.75	5 (IQR 3–13.5)	Respiratory failure	8 (IQR 2–16)	NA	61.5% mortality	46% anticoagulation
Powell et al. 2023 [25]	26 (45.6% of ECMO Population)	29 (IQR 22–40)	81%	19%	100%	0%	Average 33.4 (17)	33.40	NA	ARDS	8 (IQR 4–14)	NA	27% mortality for TBI and ECMO	Yes
Robba et al. 2017 [27]	14 (45.2% of ECMO Population)	27 (IQR 14–58)	87%	13%	VV present (unspecified)	VA present (unspecified)	Median ISS 66 (IQR 25–75)	66.00	8 (IQR 6–15)	ARDS	5.5 days (IQR 4–10; range 2–29)	1 neurosurgical intervention	7.1% mortality of patients with TBI	90% heparinization
Wu et al. 2015 [26]	5 (26.3% of ECMO Population)	40.7 (18.7)	89.50%	10.50%	47.40%	52.60%	ISS 29.0 (25–34)	29.33	NA	Severe lung injury	7.0 (4–10) days	NA	40% mortality for TBI and ECMO	84.2% heparinization
Wu et al. 2018 [57]	4 (11.1% of ECMO Population)	36 (27–49)	86.10%	13.90%	61.10%	38.90%	ISS 29 (19–45)	31.00	NA	Shock, respiratory failure	5.93 days	NA	75% mortality for TBI and ECMO	Yes, heparin-minimized protocol
Zhang et al. 2023 [16]	383 (21% of ECMO Population)	Average 35.5	84.20%	15.80%	(Weighted) 66.26%	(Weighted) 33.74%	Average ISS 34.9	34.90	NA	Respiratory/cardiac failure	8.17 days	NA	33.9% mortality for TBI and ECMO	Yes, unspecified

*AIS* abbreviated injury score, *ARDS* acute respiratory distress syndrome, *DHC* = decompressive hemicraniectomy, *ECMO* extracorporeal membrane oxygenation, *IQR* interquartile range, *ISS* injury severity score, *N*/*A* not applicable, *TBI* traumatic brain injury, *VV* veno-venous, *VA* veno-arterial

Using a standardized data extraction form, this study collected data on included study design, patient demographics, ECMO configuration, anticoagulation strategy, intracranial complication, neurosurgical interventions, and clinical outcomes. Comparative analyses were performed between institutional data and findings from the systematic review to identify consistencies or discrepancies in management and outcomes.

## Results

### Institutional Cohort

In total, seven patients with severe TBI receiving ECMO were identified. All were male, with an average age of 23.3 years. The average Glasgow coma scale (GCS) at presentation was 7.4 (range 3–14). Initial neuroimaging revealed intracranial injuries, including cerebral contusions in five (71.4%) patients, epidural hematomas (EDH) in two (28.6%) patients, subdural hematomas (SDH) in four (57.1%) patients, subarachnoid hemorrhages (SAH) in four (57.1%) patients, and diffuse axonal injuries (DAI) observed in three (42.9%) patients. The indications for ECMO included ARDS in six (85.7%) patients, acute hypoxemic respiratory failure in two (28.6%) patients, refractory hypoxemia and/or hypercarbia in three (42.9%) patients, and cardiac arrest in three (42.9%) patients. The median length of time between TBI and ECMO cannulation was 3 days (range 0–22 days). Six patients were on veno-venous (VV) ECMO, and one received veno-arterial (VA) ECMO. Anticoagulation strategies were tailored to bleeding risk. Four patients were initiated and maintained on ECMO without anticoagulation, while three patients received heparin; the average heparin bolus dose was 6667 units, and infusion was titrated to achieve an average activated partial thromboplastin time (aPTT) target of 70–100 s. Two patients (both with initial GCS 3 and no anticoagulation) underwent a decompressive craniectomy while on ECMO; one was neurologically intact at discharge, while the other died. Two patients had a craniectomy or craniotomy performed an average of 5.5 days before ECMO; one was maintained with anticoagulation while the other was not, and both were neurologically intact and had GCS of 15 at discharge. One patient underwent a decompressive hemicraniectomy (DHC) 5 days after ECMO decannulation due to interval development of an intraparenchymal hemorrhage secondary to therapeutic anticoagulation for a pulmonary embolism; they were neurologically intact and had GCS of 15 at discharge. Two patients (average initial GCS 4) did not undergo any neurosurgical procedures during the peri-ECMO period; one was on VA ECMO and anticoagulated while the other was on VV and not given anticoagulation, and both had GCS of 15 at discharge. Importantly, none of the seven patients experienced any ECMO-related hemorrhagic or thrombotic complications. All six surviving patients attained favorable functional outcomes at discharge (independent in all activities of daily living), whereas one patient died during hospitalization. The median duration of ECMO therapy was 4 days (range of 2–21 days). Table 1 provides a summary of the clinical characteristics, management strategies, and outcomes of these patients.

### Systematic Review—Case Reports and Series

With the expanding body of literature on the intersection of neurosurgery and ECMO, this study aimed to better understand the scenarios in which ECMO has been utilized for patients with neurological injuries. This study organizes the published evidence into two categories for analysis: (A) patients with TBI receiving ECMO therapy (Table 2) and (B) ECMO patients undergoing neurosurgical intervention for ECMO-related complications (Table 3). From the 43 studies meeting inclusion criteria, 26 articles were case reports or small case series, encompassing 38 individual patients relevant to the two categories.

Among the reported patients with TBI supported with ECMO, the majority were young male individuals (67.5%) with an average age of approximately 31 years. Most had severe initial neurological injury, with initial GCS scores ranging between 3 and 12 on presentation. Patients were mostly cannulated for severe respiratory failure, such as ARDS or refractory hypoxemia, and less frequently for cardiac arrest. VV ECMO was the predominant modality used for respiratory indications, while VA ECMO was typically reserved for cases with hemodynamic instability. In fact, 79% of patients with TBI on ECMO were managed with VV ECMO circuits, compared with 21% utilizing VA ECMO (Table 2).

In contrast, among the patients who required neurosurgical intervention for ECMO-related intracranial complications, only 38% were initiated on VV ECMO, with a greater majority provided VA ECMO (Table 3). These patients tended to have more complex, multiorgan involvement and polytrauma, and with longer ECMO cannulization (average duration of 11.6 days on ECMO versus 8 days for cases in Table 2). Patients with ECMO-related complications also had more patients with ECMO indications related to cardiac arrest, respiratory failure, and shock. Unfortunately, initial GCS and injury severity scores (ISS) were inconsistently reported in these cases, limiting any prognostic analysis based on those metrics.

Anticoagulation strategies varied significantly across studies, with heparin being the most used anticoagulant. Approximately 75% of the 38 cases with available data used heparin, whereas the remaining 25% either used alternative agents such as bivalirudin, followed heparin-sparing protocols, or omitted systematic anticoagulation strategies entirely. Many reports emphasized tailored dosing on the basis of coagulation profiles [e.g., activated clotting time (ACT) or aPTT)] to balance the risks of thromboembolism and ICH. Notably, some studies adopted heparin-free or minimized anticoagulation protocols to mitigate ICH risk. For example, Muellenbach et al. [9] reported a series of three patients with polytrauma ARDS with severe TBI who were managed with heparin-free VV ECMO, with none developing ECMO-related bleeding/clotting [9]. Similarly, Chen et al. [10] conducted a feasibility study of a heparin-free VV ECMO protocol as a salvage therapy for patients with trauma with contraindications to anticoagulation, achieving successful oxygenation support without ICH.

ICH complications were documented in several patients, often necessitating neurosurgical interventions and/or a reversal agent. In terms of neurosurgical intervention, decompressive craniectomy with hematoma evacuation was the most routinely performed procedure, with early intervention being associated with favorable neurological outcomes. For example, Anton-Marton et al. [5] and Bindal et al. [11] each detailed patients who suffered ICH on ECMO and underwent timely craniectomy resulting in full neurological recovery, with patients achieving GCS scores of 15 and modified Rankin scale (MRS) scores of 0 at follow-up [5]. Despite the severity of injuries and complications, favorable outcomes were achieved in a significant proportion of patients who received timely intervention, including neurosurgical when required. Although the assessment standard for patient outcomes varied among studies, patients with TBI on ECMO (Table 2) showed an approximately 95% cumulative survival (only 2 deaths among 38 patients), despite perhaps reflecting a publication bias toward successful cases. In contrast, among the ECMO-related neurosurgical complication group (Table 3), the average survival rate was 47.6%. Patients who underwent prompt neurosurgical intervention and individualized anticoagulation management often required only minimal-to-moderate assistance for their activities of daily living, with overall better functional outcomes than patients who received late intervention and standard anticoagulation management. Studies such as that of Fletcher-Sandersjöö et al. [12] reported multiple ECMO patients who had significant ICH, but with rapid neurosurgical evacuation and critical care management, some achieved full recovery or only minor deficits [12]. Common themes across these reports included early head CT imaging, close monitoring of coagulation status, and coordinated approach involving intensivists, neurosurgeons, and ECMO specialists.

### Systematic Review—Cohort and Review Studies

In addition to individual case reports, we identified 17 large studies (cohort analysis and literature review studies) that mentioned the use of ECMO in patients with trauma and specifically noted outcomes in those that mentioned at least one instance of a patient with TBI (summarized in Table 4). These studies included a wide range of sample sizes. The number of ECMO patients that had also suffered a TBI ranged from 1 to 383 (5.8–100% of the study populations) [13–16]. Among these heterogeneous populations, about 71.86% of patients were male, and median age ranged from 28 to 49.8 years [14, 15, 17–19]. Consistent with case reports, VV ECMO was the predominant modality, utilized for respiratory failure (ARDS or refractory hypoxemia). VA ECMO was utilized in fewer cases, typically only for cardiac failure [2, 10, 13, 20–22].

Anticoagulation protocols varied significantly, with protocols ranging from heparin-free approaches to low-dose or minimized heparin administration [10, 15, 18, 23–26]. Heparin-free strategies were effective in reducing the risk of ICH (Table 4). Patient-tailored, moderate anticoagulation protocols targeting lower aPTT and/or INR values to maintain circuit patency without significantly increasing the risk of hemorrhagic complications were also effective [10, 24, 26].

Reports of neurosurgical interventions among these larger studies were relatively rare but did exist. Several series noted a subset of ECMO patients who underwent decompressive craniectomies and hematoma evacuations and were associated with improved survival in select cases [11]. Across all 17 studies, mortality rates varied widely, ranging from 0% in highly selective cases to 74% in larger cohorts, with an overall average mortality rate of 30.2% [2, 12, 18, 21, 23]. Outcomes were generally influenced by the severity of the injury, with higher ISS and initial GCS scores less than 6 correlating with worse prognoses [15, 17, 19, 27].Notably, studies reported that tailored anticoagulation management, combined with early recognition of complications through imaging and timely neurosurgical intervention, was critical for optimizing outcomes. For instance, Robba et al. [26] demonstrated that proactive monitoring and intervention led to reduced mortality in patients who underwent neurosurgical intervention during ECMO [27]. Likewise, Mader et al. [21] analyzed outcomes from a large trauma database and found that although patients with TBI have historically been excluded from ECMO, the subgroup of 134 patients with TBI who received ECMO had a mortality rate of 38.1%, suggesting that ECMO may be beneficial in select TBI cases.

## Discussion

In our institutional cohort (*n* = 7), no patients experienced hemorrhagic or thrombotic complications attributed to ECMO, in contrast to the existing literature, where the incidence of ICH in ECMO patients ranges from 2 to 34% [13, 26, 28]. Five out of seven of our patients also underwent a neurosurgical procedure in the peri-ECMO period, with only one unfavorable outcome. Notably, one of our patients underwent DHC while on ECMO as a temporizing bridge to permit definitive neurosurgical treatment for ICH secondary to TBI while in acute respiratory failure [29], illustrating a practical pathway by which ECMO can stabilize physiological sufficiently to enable time-sensitive neurosurgical intervention. Although intentionally descriptive and modest in size, the cohort offers management details on a population in whom ECMO is rarely considered, and should be read alongside the broader literature rather than a stand-alone efficacy estimate.

Upon review of the existing case reports/case series, it was seen that patients with TBI on ECMO were often cannulated solely to support cardiopulmonary function and rarely received ECMO primarily to facilitate neurosurgery before an ICH occurs. Instead, those who ultimately underwent neurosurgical procedures while on ECMO typically did so because they developed critical ICH as an ECMO complication (Tables 2 and 3) [5, 11, 30]. This becomes an interesting observation when juxtaposed to the findings of our review wherein no patient with TBI was placed on ECMO solely to support their cardiopulmonary function before receiving definitive neurosurgical treatment for their ICH. All three patients with TBI who underwent neurosurgical intervention while on ECMO had developed critical ICH as a complication of ECMO, necessitating surgical intervention.

More broadly, the existing literature, including Mader et al. [21], who analyzed 12,247 patients with TBI—including 134 treated with ECMO—suggested that their observed 38.1% mortality rate indicates that ECMO does not necessarily lead to poor outcomes in this patient group, despite the risks of ICH associated with anticoagulation [20]. In fact, when dealing with patients with TBI whose primary management often revolves around ICP control, ECMO may be prudent by allowing PLV without exacerbating ICP elevations [2, 21]. This may challenge the prevailing view that TBI should be considered a contraindication for ECMO therapy. Nevertheless, clinical vigilance is essential, with frequent monitoring for neurological deterioration, particularly during the peri-ECMO period, as the risk of ECMO-related ICH is highest within the first week following cannulation [14]. Our review of neurosurgical intervention for ECMO-related complications (Table 3) shows an average survival rate of only 47.6%. This may be more indicative of poor outcomes once a complication has already occurred, rather than an inherent danger in performing neurosurgery on ECMO patients itself. The decision to operate must be undertaken on a case-by-case basis, incorporating factors such as the severity of the initial ICH, initial neurological exam, degree of deterioration once a complication has occurred, and a comprehensive risk–benefits analysis. However, since the majority still achieved favorable outcomes that would most likely not have been possible without timely neurosurgical intervention, surgery should be considered in this patient demographic when needed.

A key component of our analysis addressed anticoagulation strategies between ECMO modalities. Differences in anticoagulation requirements by ECMO modalities offer a plausible mechanistic link to intracranial risk. VA ECMO generally necessitates greater anticoagulation to prevent arterial embolization via the arterial catheter, in contrast to VV ECMO, in which the circuit can be maintained with minimal or no anticoagulation, which is particularly beneficial in patients with TBI who are often coagulopathic at baseline [31]. In our institutional cohort, six out of seven of our institutional cohort were on VV ECMO and four out of seven were not anticoagulated; among the three anticoagulated patients, the two patients with VV circuitry were provided low-dose heparin of 5000 U, while the single VA patient was given a higher 10,000 U dose (Table 1). As previously mentioned, the results of our review of case reports/case series showed that about 79% of patients with TBI on ECMO were managed with VV ECMO, while 21% received VA ECMO. In contrast, only 38% of ECMO patients who required neurosurgical intervention were started on VV ECMO, with a significantly larger proportion of VA ECMO represented. Although causal inference is beyond the scope of these data, the pattern suggests that the higher rates of VA cannulation in this latter group, along with the more aggressive anticoagulation protocols, contributed to the increased incidence of intracranial complications, subsequent neurosurgical interventions, and poorer outcomes (Table 3). Consistently, 18 of 21 (85.7%) of patients needing operative management for ICH were systemically anticoagulated during ECMO therapy (1 of 21 patient reports did not clarify the use of anticoagulation therapy). In contrast, the TBI cohort, which had a significantly greater proportion of VV ECMO cases and more conservative anticoagulation regimens, showed different outcomes (Table 2). This can be seen in cases such as Stoll et al. [32], in which low-dose 200 U heparin bolus was initiated after an initial period of 72 h VV ECMO without anticoagulation, resulting in no ECMO-related complications and good recovery, whereas both cases presented by Horio et al. [33] on VA ECMO and high-intensity 12,000 U heparinization suffered ICH, with poor neurological outcomes despite surgical intervention (death and modified Rankin score of 4–5.) Given the potential risks associated with intensive anticoagulation protocols in VA ECMO, it may be beneficial for patients with TBI, particularly those with coagulopathy, to be considered for VV ECMO with conservative anticoagulation strategies to minimize the risk of intracranial complications and improve overall outcomes. This aligns with the viewpoint of Ahmad et al. [17] that patients with TBI requiring ECMO may be safely anticoagulated by aiming for lower aPTT targets, challenging traditional beliefs.

Despite the possible risks of ECMO therapy in this patient population, they may be outweighed by the benefits of improved systemic and cerebral oxygenation. In many cases, ECMO serves as a bridging therapy, supporting patients through the critical phase of recovery from their primary pathology. However, the synergistic effects of ECMO may further extend to neuroprotection by preventing or mitigating secondary brain injury—a common consequence of cerebral hypoxia and ischemia that significantly contributes to poor outcomes in TBI [34]. Current guidelines on the management of patients with severe TBI are being updated rapidly to manage secondary brain injury in the hopes of improving outcomes in this critically ill patient population. Systemic and cerebral oxygenation are modifiable factors in a patient’s hospital course that have been shown to impact outcomes in severe TBI [34–37]. While there is no overwhelmingly strong recommendation on optimal oxygenation levels, there is evidence that hypoxemia in patients with severe TBI is associated with worse outcomes and increased mortality [34, 36, 37]. The European Society of Intensive Care Medicine Consensus makes a strong recommendation for maintaining PaO_2_ between 80 and 120 mm Hg, albeit with low level evidence [38]. Further, the Brain Trauma Foundation guidelines claim there is insufficient evidence to support advanced cerebral oxygen monitoring, but did note that hypoxia identified through advanced monitoring was associated with poorer overall outcomes [39]. Other work also corroborates the notion that decreased brain tissue oxygenation and arterial oxygenation are independently associated with worse outcomes in severe TBI [34]. These findings support the potential role of ECMO to tailor oxygenation strategies on a case-by-case basis, particularly in patients at high risk of secondary brain injury.

Another decision that clinicians must make in the event of an ECMO-related intracranial complication is whether to discontinue ECMO therapy and reverse anticoagulation prior to neurosurgical intervention. Prinz et al. [14] reported that patients who achieved normalization of coagulation profiles within the first 12 h of ICH had significantly lower mortality rates and ischemic events. As presented in Table 3, only 3 out of 21 cases were decannulated and reversed after developing ICH; all 3 had favorable outcomes with subsequent return to neurological baseline [5, 13, 40]. In other cases, patients had already been decannulated by the time of ICH; mortality among these 4 cases was 25% [12, 28, 33]. The remaining 14 cases remained on ECMO until after undergoing neurosurgery, with a 64% mortality rate. As such, the results of this study suggest that if it is possible to discontinue ECMO emergently in the case of ICH, then patients may face a better prognosis. However, this decision would require great clinical dexterity and multidisciplinary expertise, ideally with the involvement of an ECMO specialist.

## Limitations

Several important limitations must be considered when interpreting this study. First, as a retrospective single-center analysis, our institutional cohort may be subject to selection bias and incomplete case capture. It is possible that some patients with TBI who were placed on ECMO at our institution were not identified if documentation or coding did not clearly indicate the TBI diagnosis or if they were treated outside the timeframe of our record query. However, efforts to minimize this risk were made by thoroughly reviewing ECMO patient logs and trauma registries. Moreover, our analysis did not account for the severity of initial TBI using established prognostic scores, such as the Rotterdam or Marshall scores, when considering ECMO anticoagulation protocols. This omission may have limited our ability to fully assess how injury severity impacts outcomes and anticoagulation management in ECMO patients. We also did not discuss the use of EVDs or ICP monitors, both of which are not only established management procedures that can significantly improve the prognosis in patients with TBI but are also themselves invasive neurosurgical procedures associated with risks of causing or exacerbating ICH. The study’s single-center design and relatively small sample size limit the generalizability and external validity of our findings. To strengthen the conclusions, future multicenter prospective studies are recommended. Furthermore, our systematic review process was restricted to articles indexed in PubMed and Google Scholar, which may have excluded relevant studies published in other databases. Only two independent reviewers were involved in screening the articles, which could have led to inconsistencies in study selection. In many of the included review and cohort studies (Table 4), there was a paucity of detailed information on the ECMO patients with TBI. In several cases, relevant patient data were either limited to a brief mention or provided only as a few words within a large table, making it difficult to assess the specific characteristics and management strategies for these patients. This lack of comprehensive data may have hindered a more thorough understanding of the outcomes and protocols associated with ECMO in patients with TBI who are concurrently affected by severe polytrauma, thereby limiting analytic depth. In addition, we excluded other extracorporeal life support modalities other than ECMO, such as interventional lung assist, cardiopulmonary bypass, extracorporeal lung assist, and left ventricular assist devices, which may warrant future research in the TBI and neurosurgical patient population. Lastly, the potential impact of reporting bias and publication bias on our findings should be acknowledged. Patients presented in Table 2 demonstrated significantly better outcomes compared with those in Table 4, with an average mortality of 5% versus 30.2%, respectively. While this discrepancy may partially reflect a higher prevalence of polytrauma and comorbidities among patients in the latter group, it is also possible that reporting or publication bias contributed to these differences. Favorable outcomes in patients with TBI may be selectively reported and published, whereas cases involving poor outcomes, including significant morbidity or mortality, may be underreported or unpublished.

## Conclusions

This study integrates an institutional cohort (*n* = 7) with a systematic review to clarify how ECMO is being utilized in association with managing patients with TBI. In our cohort, peri-ECMO neurosurgery was feasible and associated with acceptable outcomes and without ECMO-attributed hemorrhagic or thrombotic events, while the existing literature shows that most neurosurgical procedures on ECMO occurred after ECMO-related ICH had already developed, without outcomes influenced by circuit choice and anticoagulation protocol. Across sources, VV ECMO support with conservative anticoagulation appeared preferred when clinically feasible. Successful outcomes often relied on collaborative efforts among intensivists, neurosurgeons, and ECMO specialists, as well as the application of minimally intensive anticoagulation protocols. Additionally, early neurosurgical intervention in cases of ICH contributed significantly to survival, underscoring improved outcomes in patients receiving appropriate and timely surgical management. However, discontinuing ECMO prior to neurosurgical intervention may be advantageous when clinically feasible. Collectively, our findings suggest that ECMO is not an absolute contraindication for patients with TBI when managed under vigilant oversight, challenging the traditional contraindication of ECMO in this population. As advancements in ECMO technology, such as heparin-coated circuits, centrifugal pumps, and anticoagulation-free protocols, continue to evolve and gain wider adoption, improved outcomes can also be anticipated for patients undergoing neurosurgical interventions in the peri-ECMO period. Multicenter registries and protocolized studies are needed to refine patient selection, anticoagulation targets, and decision pathways for decannulation and operative timing.
